# Multiagency approaches to preventing sudden unexpected death in infancy (SUDI): a review and analysis of UK policies

**DOI:** 10.1136/bmjph-2023-000017

**Published:** 2023-06-30

**Authors:** Helen L Ball, Alice-Amber Keegan, Daniel R Whitehouse, Louise S Cooper, Sophie R Lovell-Kennedy, Laura M Murray, Dorothy Newbury-Birch, Nicola J Cleghorn, Amanda Healy, The County Durham SUDI Project Steering Committee, Michelle Baldwin

**Affiliations:** 1 Durham Infancy & Sleep Centre, Department of Anthropoogy, Durham University, Durham, UK; 2 School of Sciences, Humanities & Law, Teesside University, Middlesbrough, UK; 3 County Durham & Darlington NHS Foundation Trust, Durham, UK; 4 Public Health, Durham County Council, Durham, UK

**Keywords:** primary prevention, community health, public health

## Abstract

**Background:**

Recent reviews of sudden unexpected deaths in infancy (SUDI) in England recommend a multiagency working (MAW) approach to prevention but lack clear guidance around how this might be implemented.

**Aims:**

In England, local authorities commission and oversee public health services. This review examines how local authority policies address implementation of MAW for SUDI prevention to understand local variations and identify strengths and weaknesses.

**Methods:**

Using a comprehensive list of all metropolitan, county, unitary councils and London boroughs in England, we systematically searched local authority websites for relevant published documents and submitted freedom of information (FOI) requests where policies or guidance for SUDI prevention had not been sourced online. We extracted data from documents using a standardised form to summarise policy contents which were then collated, described and appraised.

**Findings:**

We searched the websites of 152 council and London boroughs, identifying 36 relevant policies and guidelines for staff. We submitted 116 FOI requests which yielded 64 responses including six valid documents: 45% (52/116) of local authorities did not respond. Seventeen councils shared the same guidance under safeguarding partnerships; removal of duplicates resulted in 26 unique documents. Only 15% (4/26) of the documents included a detailed plan for how MAW approaches were to be implemented despite 73% (19/26) of the documents mentioning the importance of engaging the MAW in raising awareness of safe sleep for babies with vulnerable families. Five areas of variation were identified across policies: (1) scope, (2) responsibilities, (3) training, (4) implementation and (5) evaluation.

**Conclusions:**

There are discrepancies between local authorities in England in whether and how MAW for SUDI prevention is carried out. Strengths and weaknesses of approaches are identified to inform future development of MAW for SUDI prevention.

**WHAT IS ALREADY KNOWN ON THIS TOPIC**

- In the UK, sudden unexpected death in infancy (SUDI) now clusters in the most vulnerable families where universal provision of infant sleep safety guidance is ineffective. Local authorities (LAs) are encouraged to implement targeted multiagency working (MAW) approaches for these families.

**WHAT THIS STUDY ADDS**

- We reviewed LA SUDI prevention policies to assess where and how MAW approaches are being implemented. We found this was variable and few LAs have well-designed policies, detailed guidance or training for MAW staff.

**HOW THIS STUDY MIGHT AFFECT RESEARCH, PRACTICE OR POLICY**

- Careful consideration is needed as to which MAW staff should be involved in SUDI prevention, what their roles should be, and how those roles can be appropriately supported. MAW staff need training and clear guidance about the scope of their role in SUDI prevention. The effectiveness of targeted MAW approaches to SUDI prevention needs to be robustly evaluated.

## Introduction

 The Child Safeguarding Practice Review Panel (2020) reported that ‘in spite of substantial reductions in the incidence of sudden unexpected death in infancy (SUDI) in the 1990s, at least 300 infants die suddenly and unexpectedly each year in England and Wales’.^1^ The Review Panel’s report summarised evidence from 40 infant death cases reported in 2018, highlighting that not only do these deaths now cluster among families from deprived socioeconomic circumstances, increasingly many of the families at risk for SUDI were also at risk for a host of other adverse outcomes, including child abuse and neglect. The report noted that although universal SUDI prevention information is rigorously delivered by health professionals, many of the families most at-risk of SUDI are unwilling or unable to receive or act on this information, and that ‘something needs to change in the way we work with these most vulnerable families’ in order to prevent avoidable SUDI.^2^ Likewise the 2022 National Child Mortality Database report emphasised that 42% of unexplained deaths of infants occurred in the most socioeconomically deprived neighbourhoods.^3^ The Practice Review report authors recommended SUDI prevention should be understood as safeguarding work to include partnership working within local areas for responding to issues of neglect, social and economic deprivation, domestic violence, parental mental health concerns and substance misuse. This work, they noted, ‘needs to be embedded in multiagency working (MAW) and not just seen as the responsibility of health professionals’.^2^

Although MAW has been implemented for investigation of infant deaths since the Kennedy Report^4^ in 2004, it has only recently been applied to SUDI prevention; there is no guidance for stakeholders wishing to implement multiagency SUDI prevention strategies, and no examples of good practice exist in the public domain. At the outset of this review it was unknown whether the implementation or efficacy of multiagency SUDI-prevention approaches had been evaluated.

In February 2022, with funding from the NIHR Applied Research Collaboration for North-East and North Cumbria we began a 16-month project to design and implement a multiagency SUDI-prevention programme for County Durham, a predominantly impoverished semirural ex-mining region in the North-East of England (Index of Multiple Deprivation ranking 48/151).^5^ To inform the design of our programme, we undertook this policy review to understand the national SUDI-prevention landscape and identify good practice for informing our local programme. We report here on local authority (LA) and safeguarding partnership SUDI prevention policies and guidance documents, with emphasis on those where a MAW approach to SUDI prevention had already been implemented.

### Aim of the review

The aim of this policy review was to explore and appraise the evidence for the implementation of MAW for SUDI prevention by LAs in England to understand local variations and evaluate strengths and weaknesses.In England there 6 metropolitan county councils covering 36 local councils: Greater Manchester, Merseyside, South Yorkshire, Tyne and Wear, West Midlands and West Yorkshire, plus Greater London. There are 24 county councils covering areas known as non-metropolitan counties: Cambridgeshire, Cumbria, Derbyshire, Devon, East Sussex, Essex, Gloucestershire, Hampshire, Hertfordshire, Kent, Lancashire, Leicestershire, Lincolnshire, Norfolk, North Yorkshire, Nottinghamshire, Oxfordshire, Somerset, Staffordshire, Suffolk, Surrey, Warwickshire, West Sussex and Worcestershire. Additionally there are 58 unitary authorities which are non-metropolitan countries and districts run by a single council: Bath and North-East Somerset, Bedford, Blackburn with Darwen, Blackpool, Bournemouth Christchurch and Poole, Bracknell Forest, Brighton and Hove, Bristol, Buckinghamshire, Central Bedfordshire, Cheshire East, Cheshire West and Chester, Cornwall, County Durham, Darlington, Derby, Dorset, East Riding of Yorkshire, Halton, Hartlepool, Herefordshire, Isle of Wight, Kingston on Hull, Leicester, Luton, Medway, Middlesbrough, Milton Keynes, North East Lincolnshire, North Lincolnshire, North Northamptonshire, North Somerset, Northumberland, Nottingham, Peterborough, Plymouth, Portsmouth, Reading, Redcar and Cleveland, Rutland, Shropshire, Slough, Southampton, Southend on Sea, South Gloucestershire, Stockton on Tees, Stoke on Trent, Swindon, Telford and Wrekin, Thurrock, Torbay, Warrington, West Berkshire, West Northamptonshire, Wiltshire, Windsor and Maidenhead, Wokingham, York. There are 32 London boroughs plus City of London (metropolitan, county and unitary councils and London boroughs, individually or within partnerships).

## Methods

Three of the authors (HLB, A-AK and DRW) conducted a search of local government websites between February and June 2022, followed up in July and August 2022 with Freedom of Information (FOI) requests made to all LAs where online policies or guidance for SUDI prevention had not been sourced. For each web-search authors used the Google search engine (www.google.co.uk) and searched using the name of the location (eg, Barnsley), AND the key terms SUDI OR SIDS OR Safe Sleep OR Sudden Infant Death, AND terms specifying policy OR guidance. As the use of Boolean operators is not supported for internet searches search terms were converted into simple text strings and run using multiple word combinations (eg, ‘Lincolnshire Safe Sleep Guidance’ and ‘Lincolnshire SUDI Policy’ both find the same details at https://lincolnshirescb.proceduresonline.com/p_safer_sleep_infant.html). If no relevant documents were found via a Google search the locality website (in the format council_name.gov.uk) was accessed directly and the terms ‘SUDI’, Safe Sleep’, and ‘Sudden Infant Death’ were entered into the search bar.

This was a review of policies and guidelines rather than empirical literature, however, to ensure rigour and consistency the authors followed the PRISMA (Preferred Reporting Items for Systematic Reviews and Meta-Analyses https://www.prisma-statement.org/) guidelines as closely as possible.

### Inclusion criteria

This review focused on English policies and guidelines only. Typically, these were identified by the use of the words ‘policy’, ‘guidance’ or ‘guideline’ in the document title or introduction, however, we followed Just *et al*.^6^ in defining policies as ‘broad statement of goals, objectives and means that create the framework for activity’ and guidelines as ‘decision-support tools… designed to specify practice’ that were intended for use by members of the workforce and not explicitly targeted to families.

The eligibility criteria included any policies and guidelines aimed at LA or partner services staff. Where policies or guidelines addressed both clinical and public health domains the relevant information relating to SUDI prevention in the community was extracted. Where multiple versions of documents were sourced for a given location only the most recently dated version was included. We, therefore, excluded policies and guidelines if they: (1) were targeted primarily to parents or the public rather than staff; (2) addressed clinical settings exclusively and (3) were versions of policies that had been superseded by new updates.

### Screening

All eligible policies were included to map their geographical distribution and then screened for duplicates (undertaken by HLB, A-AK and DRW). In some cases, multiple LAs adopt an SUDI prevention policy or guidance produced by the Local Safeguarding Children Partnership or Board (LSCP or LSCB) with which they are affiliated. We found multiple duplicate documents where councils were part of the same local safeguarding partnership. In reporting on the numbers of LAs with policies or guidance we counted all LAs in the partnership, but in conducting the review, we included these documents only once (identified under the name of the partnership). Members of LSCPs were easily identified as these documents carried the logos and names of all affiliated organisations.

During screening, we found that some documents contained very similar content to those from other areas. In these cases, the producers of the document had drawn heavily on the contents of a policy or guidance from another jurisdiction, usually with acknowledgement. In this situation, a note was made of the overlap, but both the derivative documents and the originals were included in the review.

### Data extraction

A data extraction form was created via an iterative process that involved generating headings for information to be extracted from the information contained in the documents using a set of key questions (see online supplemental materials A). Data extracted included information on staff roles involved in MAW, whether training was provided to MAW staff, and what factors were used in identifying ‘vulnerable families’, how MAW was implemented and evaluated. After undertaking an initial standardisation exercise where we extracted and compared information independently from the same documents HLB, A-AK and DRW were each allocated documents to review. Each person then read each policy or guidance document in full and extracted the information relevant to this review by completing the custom extraction form. The data were then compiled into a single spreadsheet.

### Quality assessment

It is usual in conducting systematic reviews to perform a quality assessment of the results of the review. This typically involves scoring the quality of the included studies against a set of standard criteria such as AMSTAR2 (A Measurement Tool to Assess Systematic Reviews), however, as standard criteria for assessing policies and guidance documents do not exist this step could not be performed.

### Patient and public involvement

Patient and public involvement is not applicable to the conduct or dissemination of this study.

## Results

**Figure 1:**
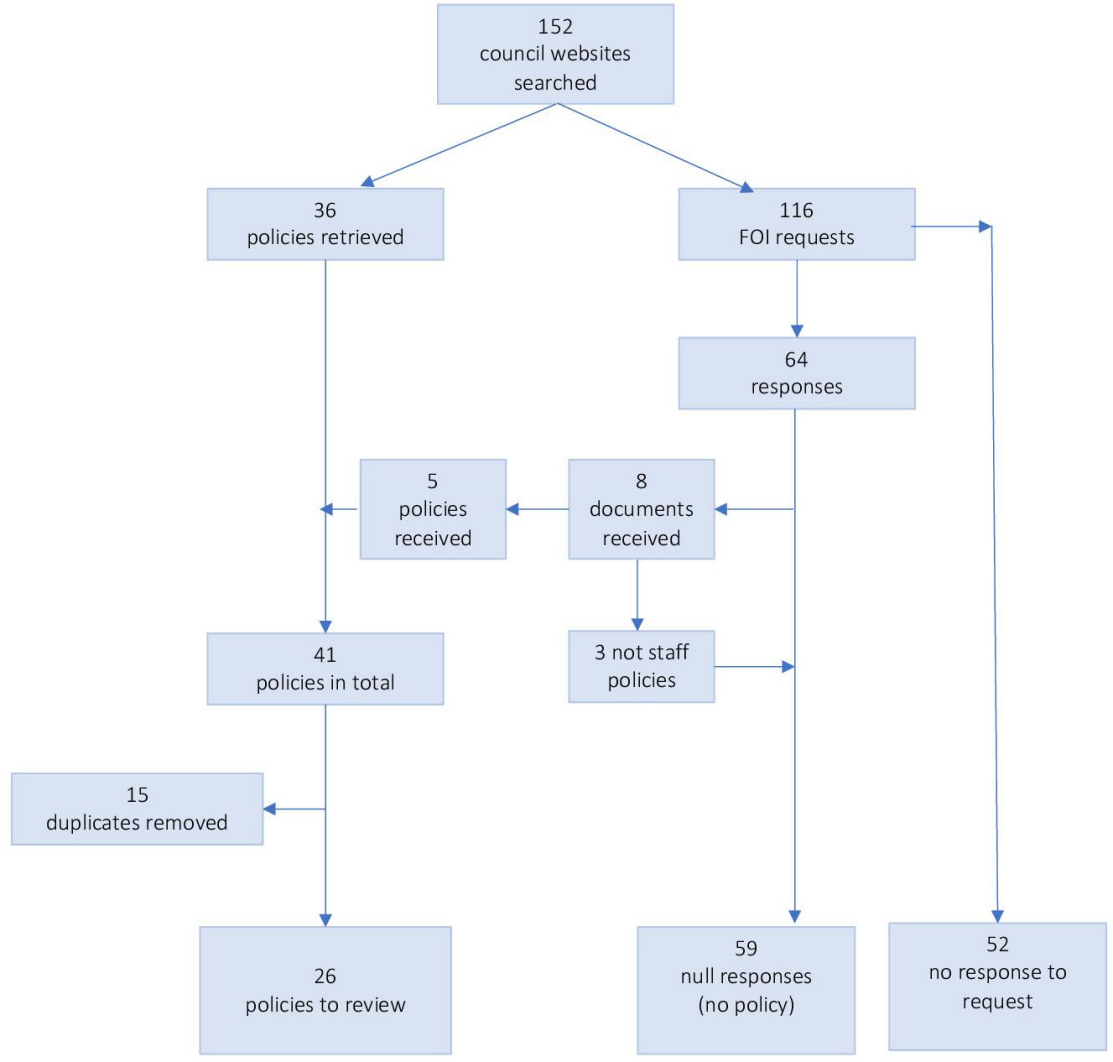
PRISMA diagram. FOI, freedom of information. PRISMA, Preferred Reporting Items for Systematic Reviews and Meta-Analyses

**Figure 2:**
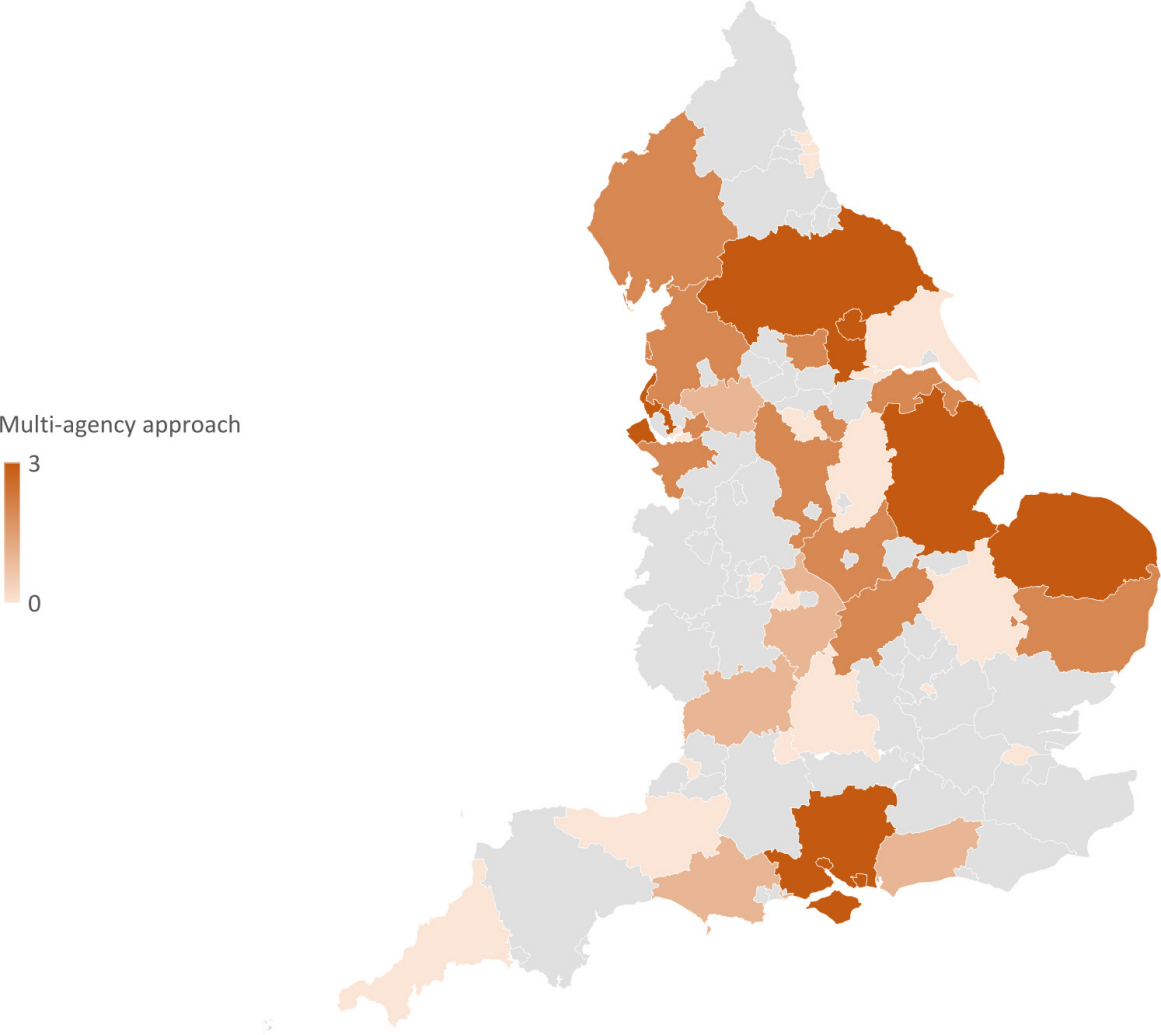
Heat map of geographic distribution of SUDI policies in England. SUDI, sudden unexpected deaths in infancy.

The document identification and screening process is summarised in figure 1. We conducted searches for 152 LAs and located 36 relevant documents meeting the search criteria. After completion of the online searches there were 116 LAs for whom no SUDI policy or guidance document had been identified. FOI requests were made to these LAs, from which 64 responses were received (55%). Eight new documents (12%) were provided via FOI responses (three were not policies and so were excluded and counted as a null response), while 56/64 (88%) authorities returned a null response (reporting they had no SUDI policy). The remaining 52/116 (45%) did not respond to the FOI request within 3 months of the request being sent. Figure 2 shows the geographical distribution of the locations from which documents were obtained.

In total SUDI policy/guidance documents for staff were obtained for 41/152 (27%) of LA. Of these, 15 councils shared guidance with one or more other councils. Three documents received via FOI requests turned out to not be policy or guidance documents, but briefings or toolkits; these were excluded. When duplicates were removed 26 remained for inclusion in the final review (see online supplemental materials B).

**Figure 3:**
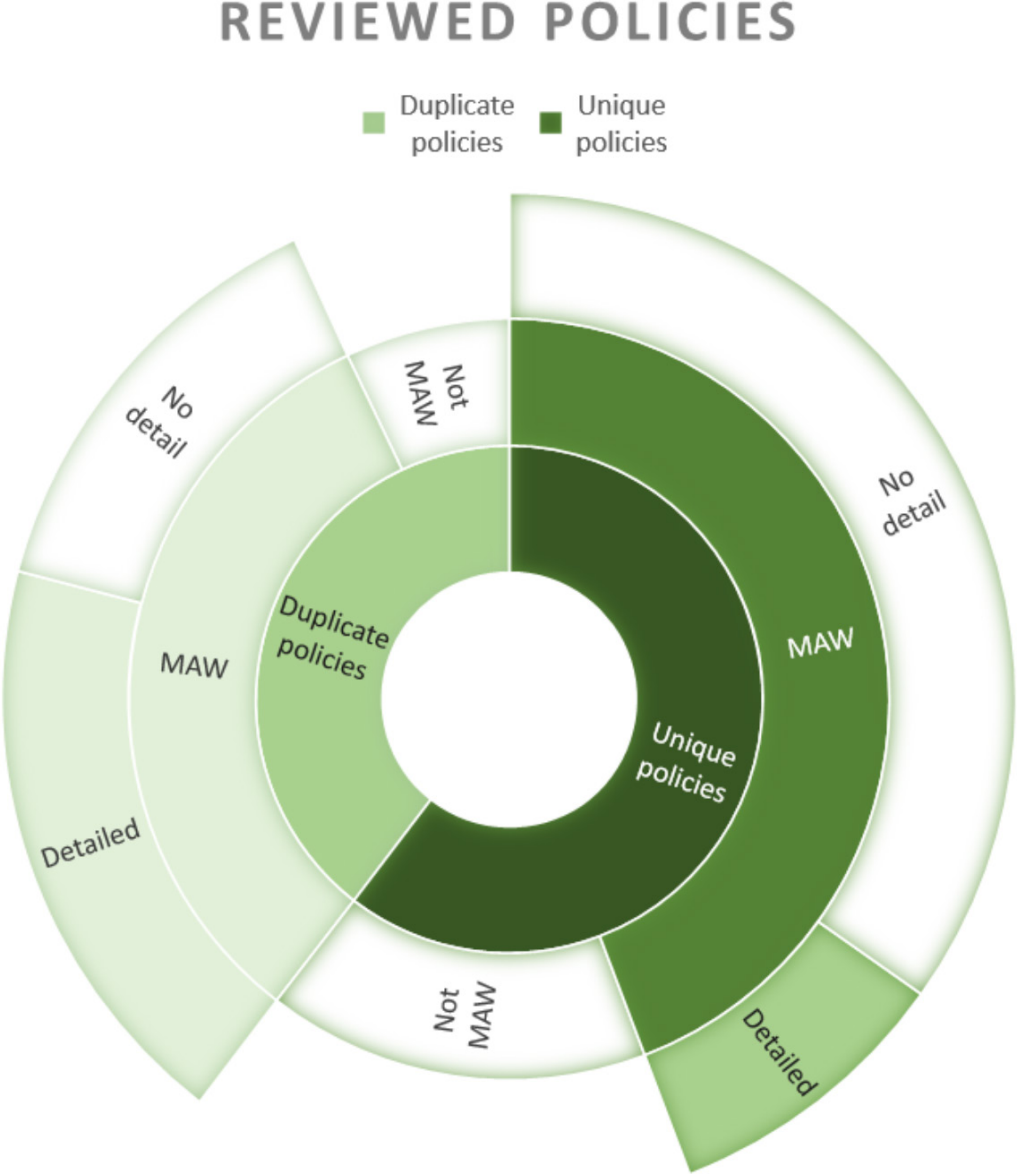
Sunburst diagram showing degree of MAW detail in policies reviewed. MAW, multiagency working.

Of the 26 policy and guidance documents reviewed 19 explicitly mentioned MAW or multidisciplinary working as a strategy for reducing unexpected infant deaths, however, only 4 of the documents discussed MAW in detail, 15 mentioned a multiagency approach but did not provide details and the remaining 7 documents did not mention MAW (figure 3).

**Figure 4:**
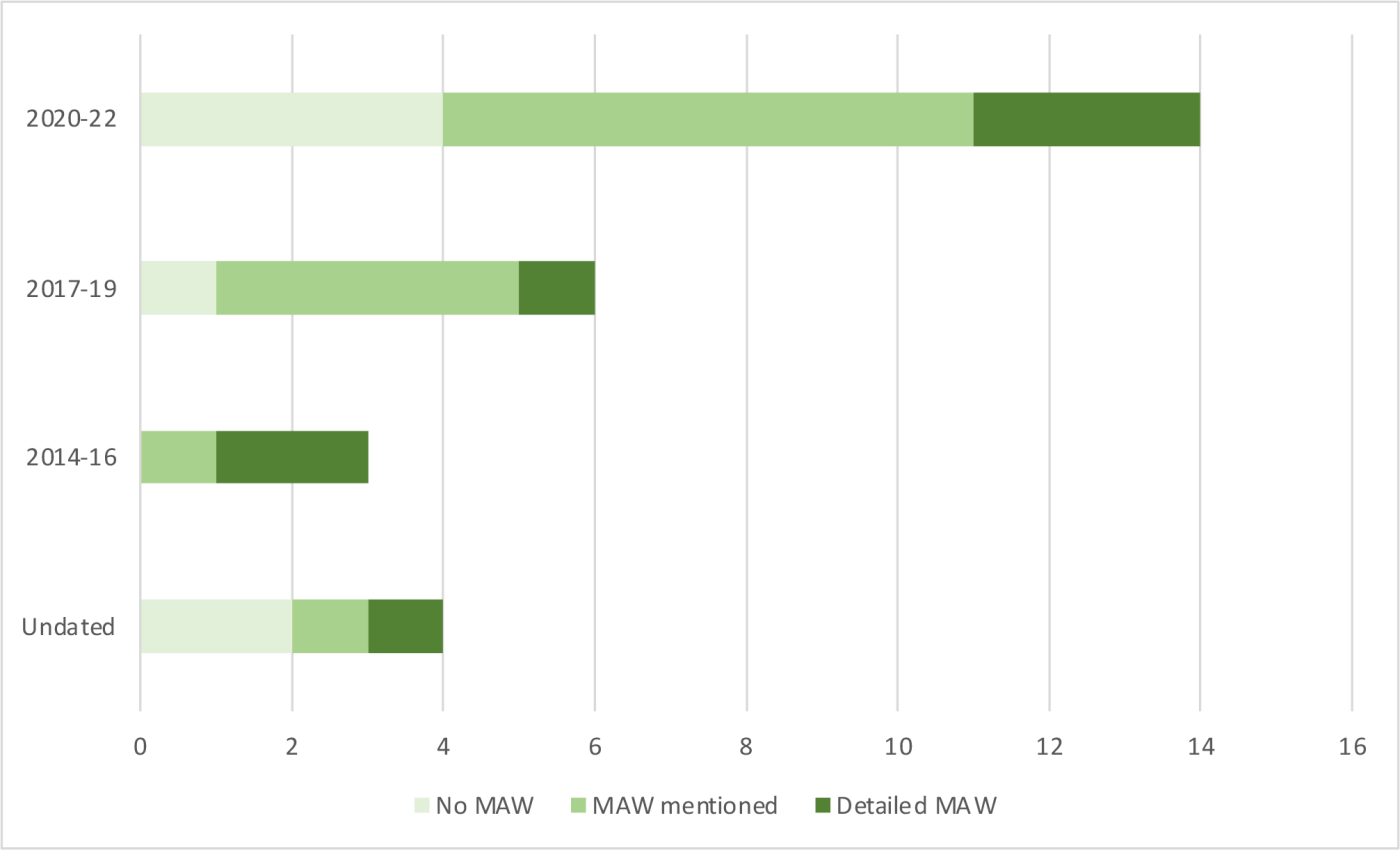
Frequency over time of multiagency working (MAW) approach mentioned and detailed in SUDI policies. SUDI, sudden unexpected deaths in infancy.

When assessed chronologically, the publication of LA SUDI policies increased over time and the inclusion of MAW in those SUDI policies also increased (see figure 4). Although MAW was mentioned in more policies, the proportion of policies including detailed MAW guidance continued to be low. There were twice as many policies published that discussed MAW between 2020 and 2022 than the preceding period, most likely in response to the publication of the Child Safeguarding Practice Review Panel (2020) report which recommended the inclusion of an MAW approach to SUDI prevention within LA settings.

### MAW approaches identified

Although it is recommended that the responsibility for SUDI prevention in vulnerable families should no longer be the responsibility solely of health professionals, only 15% of the policies and guidance documents retrieved included a detailed plan for how MAW approaches were to be implemented. This was despite 73% of the SUDI prevention policies that we examined mentioning the importance of engaging the MAW in raising awareness of safe sleep for babies with vulnerable families. No clear model of MAW for SUDI prevention emerged from this review and none of the documents reviewed indicated this approach has been evaluated for implementation feasibility or outcome efficacy.

### Staff groups included in ‘MAW’

**Figure 5:**
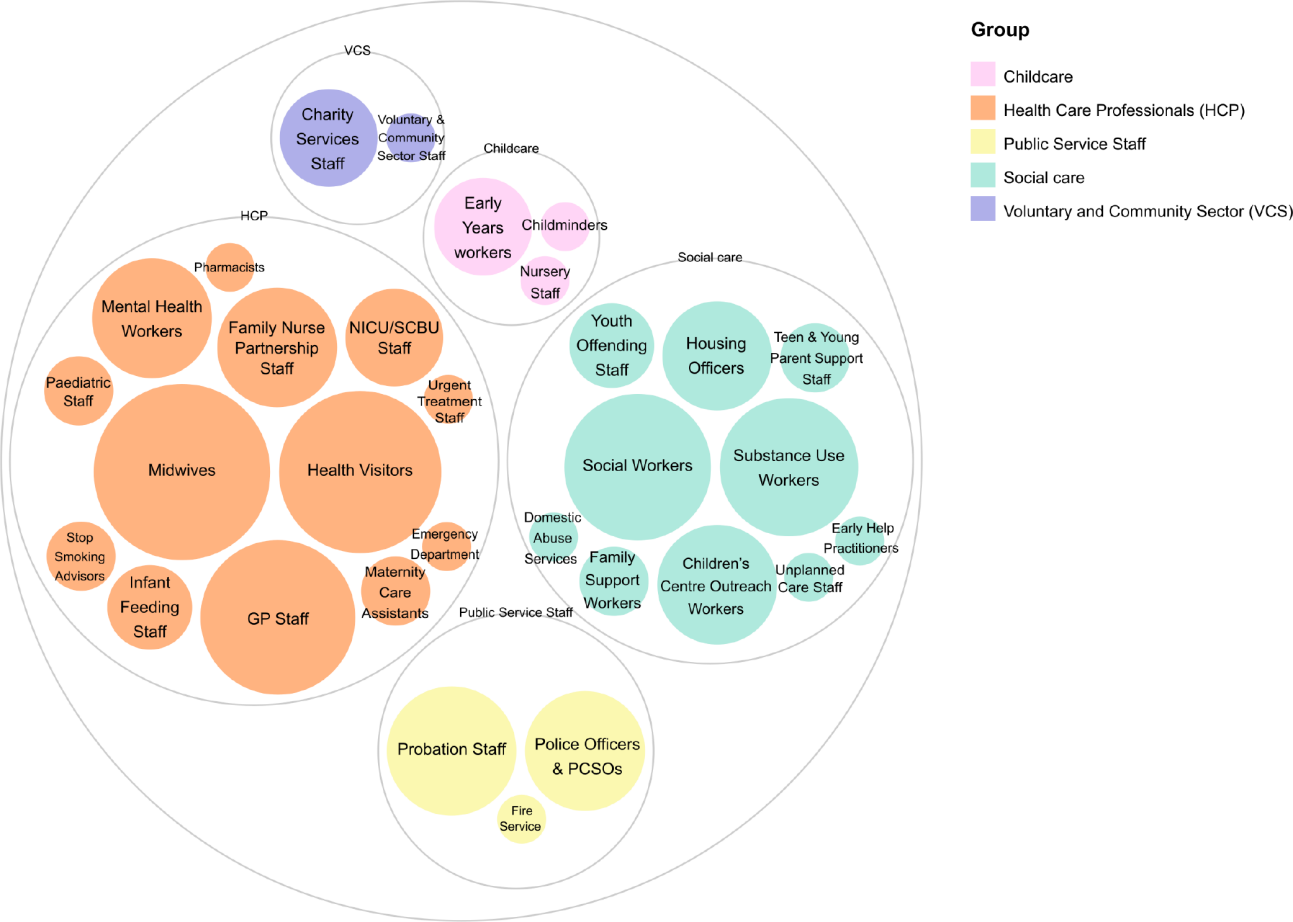
Make-up of MAW engaged in SUDI prevention. GP, General Practitioner; MAW, multiagency working; PCSOs, Police Community Support Officers; SUDI, sudden unexpected deaths in infancy.

In the 19 documents that mentioned MAW involvement in SUDI prevention 31 discrete job roles were named. Figure 5 summarises the job roles included under the umbrella of the ‘MAW’. Midwives were the most commonly mentioned occupational group involved in SUDI prevention, with 13 policies mentioning midwives, however, multiple policies specified the inclusion of non-health professional staff in SUDI prevention such as social workers, substance use workers, probation staff, police officers, housing officers and children’s centre outreach workers.

### Multiagency staff responsibilities and modes of engagement

Engagement of the MAW varied substantially across locations, and in many guidance documents MAW responsibilities lacked specificity (online supplemental materials C). In the most detailed examples (eg, Hampshire, Isle of Wight, Portsmouth and Southampton SCPB) role-specific guidance was provided for a range of diverse staff groups such as probation staff, housing officers, police officers, youth offending staff, social workers, teenage pregnancy teams, mental health workers and more. In other documents, MAW staff roles were listed but no information was given regarding the SUDI prevention responsibilities of each role. Examples of responsibilities for different MAW groups extracted from the guidance documents are illustrated in online supplemental materials C.

### Families identified as benefiting from MAW support

Across the documents discussing MAW SUDI prevention the recipients specified for MAW support were also variable, from all at-risk families mentioned in the National Safeguarding Practice Review report (eg, Barnsley SCPB) to groups of families enrolled in specialist support programmes (such as the Salford Partnership) as summarised in online supplemental materials D.

### Strengths and weaknesses of identified approaches

Few documents offered specific details for MAW involvement in SUDI prevention (either which roles, or what the scope of their involvement would be), and only two documents mentioned training provision for MAW staff.

None of the documents reviewed described the pathways, procedures or systems needed to underpin effective and joined up MAW involvement in SUDI prevention.

Although some documents referred to locally produced assessment tools or checklists no information was provided regarding evaluation or validation of these.

## Discussion

The review found familiarity with a MAW approach for SUDI prevention to be geographically widespread from the south to the north of England, with some clustering in the midlands and the north of the country. This is unsurprising as these are regions where poverty and social deprivation rates are high, and the needs of vulnerable families are pressing—therefore, such areas are likely to be early developers and adopters of any new approaches to supporting families.

In the majority of policies examined, there was an implicit assumption that relevant MAW staff would read the guidance documents, identify themselves as appropriate members of the MAW and engage in this work. However, the four detailed examples of MAW roles and responsibilities suggest that careful consideration is needed as to which members of the MAW should be involved in SUDI prevention, what their roles should be and how those roles can be appropriately supported. Public health policy-makers may need to balance the needs of their communities with the workloads of MAW in heavily used services. Additionally, MAW staff may find this work challenging and beyond their current skills, and so need training and clear guidance about the scope of their role in SUDI prevention. Although none of the documents reviewed mentioned development of the systems underpinning the policy or guidance outlined, it is likely that systems for identifying and addressing issues faced by families, as well as referral and reporting pathways may need modifying or integrating to accommodate MAW involvement, and record-keeping processes will similarly need to be considered.

Finally, engagement of the MAW in SUDI prevention also requires careful consideration of which families are identified as ‘vulnerable’, and therefore, eligible for or in need of MAW support. The documents examined in this review suggest a wide range of circumstances may be covered under the ‘vulnerable family’ umbrella; clearly defining different characteristics as contributing to ‘vulnerability’ will help specific MAW groups grasp the need for SUDI prevention training in their role (e.g. mental health workers, drug and alcohol workers, police and probation officers) but also risks stigmatising families who may notice their parenting being more closely observed.

### Limitations of the evidence

Although we can show the number and geographic spread of MAW policies and the level of detail/direction included in the policy and guidance documents reviewed, we acknowledge that this cannot be used to evaluate impact of the policies or indeed the extent to which the MAW implement them, or indeed are aware of them. We are also aware that we only obtained sight of 30% of the potential number of documents that could have been produced by LAs or safeguarding partnerships, though we have evidence from the null FOI returns that 37% of LAs had not produced one. It was disappointing that 45% of LAs contacted (34% of all LAs) failed to respond to our FOI request.

### Limitations of the review

As most of the guidance documents we retrieved were produced by local safeguarding boards we could have contacted these boards directly to request guidance documents rather than searching LA websites, however, we would have missed those that were produced independently of SCPBs by LAs and National Health Service Trusts in non-safeguarding partnerships, reducing the variability captured in this review. We also did not contact councils for whom we found out of date policies to request whether they had an updated one which was an oversight in our methods.

### Implications of the results for policy, practice and future research

Vulnerable families may avoid contact with antenatal and postnatal health professionals^7^ but are likely to encounter multiple LA and partner agency staff on a more regular basis. Making SUDI prevention everyone’s business maximises the opportunity to ensure vulnerable families are familiar with safe sleep information, and are able to implement it, with the ultimate goal of saving babies’ lives.

The strengths of the policy and guidance documents we accessed and reviewed included a clear understanding of how the MAW approach could enhance the reach of SUDI prevention information to families whose babies are most at-risk for SUDI and offered the opportunity to move beyond information provision to supporting families with, and removing barriers to, implementation. It also offers the opportunity to anticipate the needs of vulnerable families and devise specific preventative interventions.

In several cases, there was clear articulation of which MAW roles could be effectively engaged in SUDI prevention, with well-defined guidance for staff in each job role. Some guidance documents also indicated that training for the workforce had been designed and was available, and/or that MAW involvement in SUDI prevention was embedded within a local campaign around infant sleep safety (eg, Rochdale’s Keep Baby Safe or HIPS Every Sleep Counts campaigns). It is important that staff receive training to ensure they have the knowledge and confidence to initiate discussion with families around infant sleep safety.

MAW involvement in SUDI prevention is still in the early phase of implementation and it is therefore unsurprising that there were numerous weaknesses in the guidance documents produced to date. Key among these was lack of evaluation of either the implementation process or the proximate outcomes of this relatively new initiative.

## Conclusions

Although SUDI rates in the general UK population are less common now than they have been historically, it is still a significant category of death in infancy, particularly for infants born into families who are identified as vulnerable. To reduce inequalities SUDI prevention strategies must be targeted to support those most in need. This review of SUDI policies finds that there are inconsistent SUDI prevention approaches across England, with a limited number of policies explicitly mentioning a MAW approach, and considerable variation in the degree to which this is planned and executed. To develop effective MAW policies, policy-makers could aim to develop clear and comprehensive guidance on who might be involved from the MAW in any given area, and the roles and responsibilities of the MAW. Policies could also identify which families are most vulnerable to SUDI and thus who may be targeted for additional support with this approach. Guidance on implementing and evaluating the policies, processes and training that are developed should also be considered. All professionals who work with at-risk and vulnerable families can be provided with training to develop their knowledge, skills and confidence to help remove barriers to safe infant sleep and thereby prevent SUDI.

## supplementary material

10.1136/bmjph-2023-000017 online supplemental file 1

10.1136/bmjph-2023-000017 online supplemental file 2

10.1136/bmjph-2023-000017 online supplemental file 3

10.1136/bmjph-2023-000017 online supplemental file 4

## Data Availability

Data are available in a public, open access repository.

## References

[1] NHS Digital (2019). Child death reviews: year ending 31 March 2019. https://digital.nhs.uk/data-and-information/%20publications/statistical/child-death-reviews/2019/content.

[2] (2020). Out of routine: a review of SUDI in families with children at risk. https://assets.publishing.service.gov.uk/government/uploads/system/uploads/attachment_data/file/901091/DfE_Death_in_infancy_review.pdf.

[3] National Child Mortality Database Programme Thematic Report (2022). Sudden & unexpected deaths in infancy & childhood. https://www.ncmd.info/wp-content/uploads/2022/12/SUDIC-Thematic-report_FINAL.pdf.

[4] Royal College of Pathologists (2004). Sudden unexpected death in infancy: a multi-agency protocol for care and investigation.

[5] (2019). 8 index of deprivation 2019 - summary for County Durham. https://democracy.durham.gov.uk/documents/s119825/8%20Index%20of%20Deprivation%202019%20-%20Summary%20for%20County%20Durham_cabinet.pdf.

[6] Just D, Tai S, Palmier-Claus J (2023). A systematic review of policy and clinical guidelines on positive risk management. J Ment Health.

[7] Redshaw M, Heikkila K (2010). Delivered with care: a national survey of women’s experience of maternity care.

