# Supplementary material for: Multiagency approaches to preventing sudden unexpected death in infancy (SUDI): a review and analysis of UK policies
Source: BMJ Public Health. 2023 Jun 30;1(1):e000017. doi: 10.1136/bmjph-2023-000017 (PMC11812686; doi:10.1136/bmjph-2023-000017)
Supplement: online supplemental file 1 [file bmjph-1-1-s001.pdf]

Supplementary Materials A

Key questions re. SUDI policies guiding creation of data extraction form

Question	Information captured
Who produced policy/guidance?	Local authority, safeguarding partnership, local NHS-council partnership
What geographic area does it cover?	Name of city, county or urban conurbation
Document title and date	Title details
How was document sourced?	Online, FOI, other
Is document available online?	URL where relevant
What is the document type?	Policy, staff guidelines, recommendations, toolkit
What is the key aim or purpose?	Key aim or purpose cut and pasted verbatim
Were key terms defined?	Were definitions given for SUDI, SIDS, bed-sharing, co-sleeping etc.
Was safer sleep messaging given?	List of key messages or reference to messaging produced by another source (e.g. Lullaby Trust, NHS).
Was multi-agency working (MAW) or multi-disciplinary working (MDW) for SUDI prevention discussed?	Yes, discussed in detail.<br>Mentioned but no details given.<br>Not discussed.
Who was specifically identified within the MAW/MDW?	List all job roles mentioned
Were MAW/MDW responsibilities specified	Yes, responsibilities were differentiated;<br>Yes, all staff had the same responsibilities;<br>No, responsibilities were not specified.
Was SUDI guidance universal (one-size-fits-all) or targeted?	Universal,<br>Targeted,<br>Both,<br>Unspecified,<br>Unclear.
Does the document reference families needing extra support (e.g. vulnerable families)?	Yes<br>No
Which families are specified as 'vulnerable'	List all factors specified e.g. substance abuse, domestic violence etc.
Was a referral pathway identified for further support for families	Yes<br>No<br>Unclear.
Was SUDI prevention embedded within child safeguarding?	Yes<br>No<br>Unclear.
Were parent tools identified	Yes<br>No.
What does this document provide good information on?	Give summary of strengths
What does this document not address?	Give summary of weaknesses
