# Supplementary material for: Multiagency approaches to preventing sudden unexpected death in infancy (SUDI): a review and analysis of UK policies
Source: BMJ Public Health. 2023 Jun 30;1(1):e000017. doi: 10.1136/bmjph-2023-000017 (PMC11812686; doi:10.1136/bmjph-2023-000017)
Supplement: online supplemental file 2 [file bmjph-1-1-s002.pdf]

Supplementary Material B: Table of included policy / guidance documents

Local authority / safeguarding partnership	Date	Document title	How sourced	Multi-agency approach?	MAW roles specified?
Barnsley Safeguarding Children Partnership	2022	Prevention of Sudden Unexpected Death in Infancy Multi Agency Guidance	FOI	Mentioned	Yes
Coventry/Warwickshire Safeguarding Children Partnership	2021	Safer Sleeping: A Guide for Practitioners	Web	No	NA
Cumbria Safeguarding Children Partnership	2021	Cumbria Safer Sleeping Guidance for Children	Web	Mentioned	No
Derby & Derbyshire Safeguarding Children Boards	2021	Partnership Strategy to Support the Safety of Babies in Derby and Derbyshire	Web	Mentioned	No
Dudley / Black Country Partnership	2018	Dudley Safer Sleep Policy	Web	No	NA
Gloucestershire Health & Care NHS Trust	2021	Sudden Infant Death - A brief guide for Professionals	Web	No	NA
Hampshire & Portsmouth Safeguarding Partnership	Nd	Safe Sleep for Babies and Infants	Web	Detailed	Yes
Kingston & Richmond Safeguarding Children Boards	2022	Baby and Infant Safe Sleeping Practice Guidance	Web	No	NA
Leicestershire Partnership NHS Trust	2019	Safer Sleeping and Reducing the Risk of Sudden Infant Death Syndrome Guidelines	Web	Mentioned	No
Lincolnshire Safeguarding Children Partnership	2021	LSP Policy and Procedures Manual: Safer Sleep for Infants Guidance	Web	Detailed	Yes
London Borough of Sutton	2022	London Borough of Sutton Practice Directive (for babies in care)	FOI	Detailed	NA
Manchester Safeguarding partnership	2021	Safer Sleeping Practice for Infants	Web	No	NA
Merseyside Safeguarding Children Partnership	2017	Multi-Agency Safe Sleeping Guidance	Web	Mentioned	No
Norfolk Safeguarding Children Partnership	2022	Strategy for Protecting Babies from Harm	Web	Mentioned	Yes
Northamptonshire Safeguarding Children Board	Nd	Safer Sleep Guidance for Partner Agencies	Web	Mentioned	Yes
North Lincolnshire Partnership	2021	Joint Safe Sleeping Guidance A resource for all organisations	Web	Mentioned	Yes
North Yorkshire & York Safeguarding Children Boards	Nd	Day or night, sleep right. Preventing SUDI: multi-agency risk minimisation guidance	Web	Mentioned	No
Pan Cheshire Multi-Agency Services	2019	Pan Cheshire Infant Safe Sleep Guidance for the Integrated Workforce	Web	Mentioned	No
Pan Dorset Safeguarding Children Partnership	2022	Safer Sleeping Guidance for Practitioners to Support Parents/Carers	FOI	No	NA
Pan Lancashire Safeguarding Children Board	2020	Safer Sleeping Guidance for Children	Web	Mentioned	No
Rochdale Safeguarding Board	2022	Rochdale Safe Sleep for Infants Multi-Agency Guidance	Web	Mentioned	No
Rotherham Safeguarding Children Partnership	2017	Safe Sleeping for Infants Guidance	Web	Mentioned	No
Salford Safeguarding Children Partnership	2021	Salford Safer Sleep Guidance for Children	Web	Detailed	Yes
Suffolk Safeguarding Children Board	2018	Safer Sleep Guidance for Partner Agencies	Web	Mentioned	Yes
West Sussex Safeguarding Children Partnership	Nd	Working with Families: Safe Sleeping	Web	No	NA
West Yorkshire Health & Care Partnership	2022	Every sleep a safe sleep: Multiagency risk minimisation guidance	Web	Mentioned	No
