# Supplementary material for: Multiagency approaches to preventing sudden unexpected death in infancy (SUDI): a review and analysis of UK policies
Source: BMJ Public Health. 2023 Jun 30;1(1):e000017. doi: 10.1136/bmjph-2023-000017 (PMC11812686; doi:10.1136/bmjph-2023-000017)
Supplement: online supplemental file 3 [file bmjph-1-1-s003.pdf]

Supplementary Material C: Example specifications of SUDI prevention responsibilities for different roles within the MAW/MDW

Barnsley Safeguarding Children Partnership (2022)

All practitioners & workers

- All practitioners and workers are expected to be able to provide the same consistent message in relation to safe sleeping and to challenge any factors that contribute to unsafe sleeping. Unsafe sleep practices may be observed in any environment, home, office or public space and at any time of the day or night. On each and every occasion of an observed unsafe sleep practice workers and practitioners must be able to offer current evidence based information enabling the parents to recreate the sleep space and safeguard against SUDI.

Midwifery, Public Health Nursing, Children's Services Staff, Social Workers, Community Neonatal Nursing

- Identification of the broader continuum of risks associated with SUDI; Undertaking collaborative safe sleep risk assessments with the family; Co-producing safe sleep action plans with the family;
- Identify those families who require personalised and targeted additional support and refer to services as appropriate; Motivational Interviewing approach to supporting safe sleep practices.

Police; Probation; Housing; Fire service; Drugs and alcohol services, Smoking cessations services

- Workers should ensure that they are alert to the infant's sleep space and make every contact count.
- All workers should be able to support parents maintain a safe sleep environment for their infants through the provision of key safe sleep messages, enabling parents to understand SUDI risk factors and understand how to escalate concerns about unsafe infant sleeping practices.

Hampshire, Isle of Wight, Portsmouth & Southampton (undated)

Midwifery

- Discuss what has been purchased/sourced for the baby's sleeping arrangements, i.e. cot, Moses basket, bedding and provide
- support for them to access financial aid if needed.
- Following delivery, the same universal safer sleeping method applies – the safest place for the baby to sleep is in a cot/Moses
- basket, alongside the parent's/carers bed.
- The Midwife should document that safer sleep messages and arrangements have been discussed with expectant families within
- the Personal Child Health Record (red book).
- Advice should be offered to address any apparent risk factors and ensure all advice regarding protective factors is clearly
- communicated. Any risk factors which have been identified and the subsequent action plan should be documented.
- They should discuss the sleeping arrangements for the baby/infant with all known carers, including the father, grandparents, etc.

Health Visiting and Family Nurse Partnership

- At the antenatal contact, the Health Visitor should discuss with parents/carers their plans for sleep arrangements for their new
- baby and begin to introduce the safer sleeping messages. This should be documented in the Parent Held Record if available.
- Check that they have a cot/Moses basket and provide support for them to access financial aid if needed.
- They should discuss the sleeping arrangements for the baby/infant with all known carers, including the father, grandparents, etc.
- At the new birth visit, the Health Visitor should revisit the safe sleep messages. This should be combined with a discussion on
- sleep routines during the day and night.
- At the postnatal check, repeat as in new birth visit and clarify that safe sleep arrangements are in place. Should the parent choose to decline to follow the advice, this must be documented.
- For Family Nurse Partnership teams (FNP), discussions as outlined above should take place during FNP pregnancy visits and
- revisited during postnatal contacts, agenda matched with the client.

General Practitioners/All Practice Staff

- GP's and practice staff should be familiar with the safer sleeping messages and practice guidance and should encourage parents/carers of new babies and young children to be aware of safer sleep publicity materials
- GP's and practice staff who have consultations with pregnant women, their partner and parents of new or very young babies should use the opportunity to ask about sleeping arrangements for their baby and promote safer sleeping messages, highlighting the risks and protective factors.
- GP's or other health professionals who undertake the 6-8 week postnatal health review should ask about sleeping arrangements for the baby and promote safer sleeping messages, highlighting risk and protective factors
- GP's and practice staff should be mindful when prescribing medication that may cause drowsiness or other associated impairment to a parent of a child under the age of 3 years, in line with the offence of overlay.

Mental Health Services

- When working with a family with a child less than 12 months of age in the household, mental health workers should discuss and promote the safer sleeping message.
- They should discuss the sleeping arrangements for the baby/infant with all known carers, including the father, grandparents, etc.
- Check that they have a cot/Moses basket and provide support for them to access financial aid if needed.
- Ask the parent whether the baby sleeps in other places during the day, offering safer sleeping advice where appropriate e.g. not to be placed on the sofa.
- They should routinely promote the message that the safest place for infants to sleep is in a cot /Moses basket in the same room as their parents for the first six months
- Ask what arrangements are in place if the parent is taking prescribed medication for a mental health problem which may make them drowsy or sedated and could impact on their responsiveness or awareness.
- Explore what arrangements they make for the baby if they choose to drink alcohol and/or take drugs as well as their prescribed medication.
- Discuss the risks of sedation associated with medication, drugs and alcohol and the need to be particularly mindful at these times as to the risk of falling asleep with the baby.
- Reinforce that clients should not co-sleep or share a bed, sofa or armchair with a baby.
- Share information about discussions with the parent and any safer sleeping issues that have been identified with other workers involved with the family as appropriate
- Record all discussions clearly on service user's Ule as safer sleeping advice given and highlight any risk factors that the service user states they are to continue practicing
- Document any advice that was given in cases where a service user is experiencing mental health problems and/or uses alcohol or substances and is pregnant
- The mental health worker needs to discuss what plans the parents have and what have they purchased/sourced for their baby to sleep in. Liaise with other agencies if financial support is needed to purchase essential items.
- Liaison with other professionals who are involved with the family should be considered where appropriate.

Substance Misuse Services

- When working with a family with a child less than 12 months of age in the household, substance misuse workers should discuss and promote the safer sleeping message.
- They should discuss all points as above.
- Record all discussions clearly on the service user's Ule as to safer sleeping advice given and highlight any risk factors that the service user states they are to continue practicing and what advice was given.
- Liaison with other professionals who are involved with the family should be considered where appropriate

Infant Feeding Workers (IFW)/Maternity Care Assistants (MCA)

- Check the pregnant woman has received materials from her Midwife. If not, this should be referred to the local midwifery team or the Health Visiting Team.
- Ask if the midwife discussed Safer Sleep after the mother and baby left the place of delivery. If not, this should be referred to the Midwifery team
- If the Midwife has not yet visited, the IFW/MCA should remind the parent(s) of the key messages
- Ask the parent to talk to other people who care for a baby about the safety measures
- Discussions should be documented in the Personal Child Health Record (red book)
- At breastfeeding support groups or baby's first food groups, the safer sleeping messages should be reinforced
- During brief telephone contacts it may not be appropriate to raise the issue of safer sleeping. However, during any phone

- discussion about managing the night feeds or baby's sleep patterns, parents should be reminded about the key message on safer sleeping.

Breastfeeding Volunteers

- They should support the consistent safer sleeping messages in their work in breastfeeding support groups, antenatal sessions
- and any other work place.
- If they identify that a parent is unclear about the messages, they should speak to the Midwifery or Health Visiting team.

Children's/Family Centres and Outreach Staff

- Discuss safer sleep messages with parents/carers and signpost to appropriate health services as required
- If any parent/carer is known to be using substances and/or alcohol, ask what arrangements they make for the baby to ensure their safety if they are going to drink or take drugs. Highlight the specific risks regarding bed-sharing when under the influence of alcohol, drugs and if they smoke.

Social Workers/Social Work Assistants

- When Social Workers are undertaking an assessment and there is an infant under 12 months in the home, safe sleep messages
- as outlined in this procedure should be discussed with the parent/carers.
- If parent/carer is pregnant, advice should be given about how the future parent can access financial support to purchase a Moses basket/cot, such as government grants, if unable to purchase by their own financial means
- Continue the discussion to highlight other safety measures and explore any risk factors and what action needs to be taken to
- reduce risk; identify with all the adult carers in the home, including male carers, what practical steps can be taken to reduce risk
- Highlight the specific risks regarding bed-sharing when under the influence of alcohol, drugs and if they smoke; be very clear that under no circumstances when they are under the influence of alcohol and/or drugs should they sleep with their baby in bed or on a sofa or armchair, and that the baby should be placed in a cot/Moses basket, which is of a size suitable to the baby with appropriate bedding, giving the baby room to breathe to ensure the baby cannot suffocate or overheat

Other Agencies

It is agreed by the partnership that the ongoing promotion of safe sleep messages should be undertaken by all agencies that come into contact with children and families. This may include signposting and visual promotion, i.e. posters and leaflets.

Police

- Police officers and staff attending incidents or visiting addresses should ensure that safeguarding of children is paramount.
- In line with Voice of the Child- for residing children under 12 months they should, where practical and appropriate, establish where the infant sleeps and consider whether the environment follows the HIPS safer sleep advice.
- When safer sleep risk factors have been identified or are suspected (co-sleep, alcohol/ drug use, sofa sleeping) they will signpost parents/ carers to advice on the Lullaby Trust/ Hampshire leaflet.
- A record of this interaction and the potential risk should be submitted to MASH via a PPN1.
- This policy does not alter or in any way replace the power for Police Constables under S46 of the Children Act 1989 to remove a child where there is reasonable cause to believe the child would otherwise be likely to suffer significant harm.
- Professionals should consider explaining the criminal offence of Overlay; this relates to the cause of death for an infant less than 3 years of age being due to suffocation (not caused by disease/ foreign body). For this offence criteria to have been met, the infant must have been in bed with a person who has attained the age of 16 years when that person was under the influence of drink or a prohibited drug either when they went to bed or at any later time before the suffocation (includes any kind of furniture or surface used for sleeping).

Probation

- All probation staff working with individuals/families who have a child under 12 months of age should discuss safer sleeping
- arrangements and record accurately what was said and to whom.
- Staff should share information about what was discussed and any safer sleeping issues that have been identified with other

- professionals involved with the family where appropriate.

Housing Officers/Agencies

- Use any assessment tools, including during home visits, to identify any safer sleeping risk factors, such as drug/alcohol use, the baby is sleeping in a car seat, or is seen sleeping in a situation that does not follow the safer sleeping advice contained within this guidance
- Share concerns with the parent/carer and work to support the parent/carer to access support from other professionals involved to make safer sleeping arrangements.
- When supporting expectant families, housing staff should look to support the provision of an environment that facilitates parents being able to follow safe sleep messages, considering the facilities available or ensuring that accommodation is appropriately adapted to allow for safe sleep to take place.

Education/Early Years settings

- Follow and practice safe sleep arrangements for those babies/children who are left in the care of the early years setting.
- If discussions with parents/carers identifies concerns regarding sleeping arrangements at home, to discuss and explore safe sleep messages with parents and liaise with other professionals where appropriate and with consent where appropriate.
- Actively promote safe sleep messages, including visual promotion with leaflets and posters.

Acute hospitals, Urgent Treatment Centres etc

- If discussions with parents/carers identifies concerns regarding sleeping arrangements at home, to discuss and explore safe sleep messages with parents and liaise with other professionals where appropriate and with consent where appropriate.
- Actively promote safe sleep messages, including visual promotion with leaflets and posters.

Lincolnshire Safeguarding Children Partnership

Midwives; Health visitors; Early Help Workers; Early Years Workers; Social Workers; Child minders; Nurseries and other early years settings; Family support volunteers or peer supporters.

For services working directly with families, this policy recommends that:

- Practitioners are aware of this policy, know the key messages and receive safer sleep training;
- Safer sleep training is incorporated into the practitioners' training programme and CPD;
- Practitioners discuss safer sleeping arrangements with families, and their wider support network, at each opportunity;
- Practitioners have access to safer sleep resources that can be used with/given to families;
- Safer sleep is incorporated into the appropriate service operational guidance and procedures, or risk assessment tools;
- Safer sleep discussions and actions are documented;
- Agencies promote safer sleep message through their communication channels;
- For settings where infants are put to sleep, safer sleep is incorporated into its relevant policies and risk assessments.

Housing teams; Mental health support services; Emergency services; Carers; Pharmacists; GP practices; Probation service; Adult services (Learning Disabilities Team); Domestic abuse services; Drug and alcohol services.

For services that have brief contact with families but may observe unsafe sleeping practices, this policy recommends that:

- Staff have training to understand the principles of safer sleep and why this relates to their area of work;
- Staff understand that infants are at increased risk when there is a change of circumstances (which is the likely reason the service has got involved);
- Safer sleep is incorporated into the appropriate service operational guidance and procedures, or risk assessment tools;
- Staff know how to complete the Early Help assessment tool which assists them to support families and encourage open discussions.

Some services require specific guidance:

Neonatal nurses. When babies are preterm or very sick, they may be positioned on their front, side or back and aids to support their bodies such as positioners may be used. Neonatal Unit staff discuss safe sleep with parents while their baby is on the Neonatal Unit and also prior to discharge from the Neonatal Unit. These discussions include highlighting the particular risks of co sleeping when the baby is preterm or low birth weight. Parents are provided with the Lullaby Trust Safer sleep advice prior to discharge both in paper format and available electronically via a QR code linking to maternity postnatal leaflets webpage.<br><br>*Fostering and adoption services* should be aware of the increased risk of SIDS when a parent has smoked or consumed drugs or alcohol in pregnancy. This should be part of the risk assessment process and discussed with the fostering/adopting family.<br><br>*Smoking cessation service* should be aware of the association between smoking and SIDS and ensure this is communicated to the family.
Pan-Lancashire Children's Safeguarding Board<br><br>*Children & Family Wellbeing Service, Primary Care, Paediatrics, Emergency Department or Social Care.*<br><br>- It is the responsibility of the multi-disciplinary workforce to discuss and record, in line with record keeping guidelines, the information they give to babies' carers on safer sleeping arrangements at all key contacts including asking to see where baby sleeps.<br>- Information must be provided in such a manner that it is understood by the baby's carer. For babies' carers who do not understand English, an approved interpreter should be used where possible, appropriate and available. Families with other language and communication needs, including learning disabilities, should be offered information in such a way to ensure understanding.<br>- If any additional needs are identified the practitioner should complete the Keeping Infants & Babies Safe; Review of Parental Awareness Tool (see appendix 4).<br>- Anyone in contact with families should take every opportunity to discuss safer sleeping arrangements for babies and highlight best practice recommendations as well as risks based on current evidence.
Rotherham Safeguarding Children Partnership<br><br>*Responsibilities of the Multi-Disciplinary Workforce*<br><br>- It is the responsibility of the multi-disciplinary workforce to discuss and record, in line with record keeping guidelines of their employing organisation or professional bodies, the information they give to baby's carers on safer sleeping arrangements at all key contacts.<br>- Information must be provided in such a manner that it is understood by the baby's carer. For those babies' whose carers do not understand English, an approved interpreter should be used where possible and appropriate. Families with other language and communication needs, including learning disabilities, should be offered information in such a way to aid understanding.<br>- Anyone in contact with families should take every opportunity to discuss safer sleeping arrangements for babies and highlight best practice recommendations and risks based on current evidence. This should include safety information that encourages parents to 'think carefully about their baby's safety for every sleep' (ISIS 2015).
