# Supplementary material for: Multiagency approaches to preventing sudden unexpected death in infancy (SUDI): a review and analysis of UK policies
Source: BMJ Public Health. 2023 Jun 30;1(1):e000017. doi: 10.1136/bmjph-2023-000017 (PMC11812686; doi:10.1136/bmjph-2023-000017)
Supplement: online supplemental file 4 [file bmjph-1-1-s004.pdf]

Supplementary Materials D: Examples of families identified as needing extra support or resources from MAW

Location	Families identified for MAW support
Barnsley	Families who experience socio-economic deprivation, living in poor or overcrowded accommodation, parents with adverse childhood experience, parents with reduced ability to detect harm in their own interpersonal relationship, parental mental health problems, alcohol or substance misuse, ongoing and cumulative neglect, parental criminal behaviours, relationship breakdown and/or new partners
Manchester	Homelessness/transient lifestyle/inappropriate housing plus any one of the following stresses: mental illness, domestic abuse, drug or alcohol use, probation/criminal justice team, social exclusion, parental hearing impairment.<br><br>- Substance misuse which raises concerns around safe and consistent parenting and/or has the potential to place the baby at risk.<br>- A previous unexplained death of a child in the family.<br>- A violent criminal history against a child or partner.<br>- Parents who have experienced a difficult childhood<br>- Late booking for ante-natal care (no proof of care before 22 weeks).<br>- Previous child not living with a parent.
Salford	Families accessing support from Early help and Family Nurse Partnership
West Yorkshire	Families including unsupported younger parents, smokers, alcohol users, illegal drug use, excessive tiredness and use of prescribed medication, mental ill health
